# Three-arm phase II trial comparing camrelizumab plus chemotherapy versus camrelizumab plus chemoradiation versus chemoradiation as preoperative treatment for locally advanced esophageal squamous cell carcinoma (NICE-2 Study)

**DOI:** 10.1186/s12885-022-09573-6

**Published:** 2022-05-06

**Authors:** Yang Yang, Li Zhu, Yan Cheng, Zhichao Liu, Xiaoyue Cai, Jinchen Shao, Ming Zhang, Jun Liu, Yifeng Sun, Yin Li, Jun Yi, Bentong Yu, Hongjing Jiang, Hezhong Chen, Hong Yang, Lijie Tan, Zhigang Li

**Affiliations:** 1 Department of Thoracic Surgery, Shanghai Chest Hospital, Shanghai Jiao Tong University, 241 West Huaihai Road, Shanghai, 200030 China; 2 Department of Radiology, Shanghai Chest Hospital, Shanghai Jiao Tong University, Shanghai, China; 3 Department of Radiation Oncology, Shanghai Chest Hospital, Shanghai Jiao Tong University, Shanghai, China; 4 Department of Integrative Medicine, Shanghai Chest Hospital, Shanghai Jiao Tong University, Shanghai, China; 5 Department of Pathology, Shanghai Chest Hospital, Shanghai Jiao Tong University, Shanghai, China; 6 Department of Thoracic Surgery, Cancer Hospital Chinese Academy of Medical Sciences, Beijing, China; 7 Department of Cardiothoracic Surgery, Jinling Hospital, Medical School of Nanjing University, Nanjing, China; 8 Department of Thoracic Surgery, The First Affiliated Hospital of Nanchang University, Nanchang, China; 9 Department of Thoracic Surgery, Tianjin Medical University Cancer Hospital, Tianjin, China; 10 Department of Thoracic Surgery, Changhai Hospital Affiliated to The Second Military Medical University, Shanghai, China; 11 Department of Thoracic Surgery, Sun Yat-Sen University Cancer Center, Guangzhou, China; 12 Department of Thoracic Surgery, Zhongshan Hospital Affiliated to Fudan University, Shanghai, China

**Keywords:** Esophageal cancer, Camrelizumab, Chemoradiotherapy, Chemotherapy, Clinical trial

## Abstract

**Background:**

Preoperative chemoradiotherapy (CRT) with CROSS regimen has been the recommended treatment for locally advanced esophageal squamous cell carcinoma (ESCC). The addition of programmed cell death protein 1 (PD-1) inhibitor to preoperative CRT may further improve oncologic results. Preoperative camrelizumab plus chemotherapy has been demonstrated as a promising treatment modality based on results of the phase II NICE study (ChiCTR1900026240).

**Methods:**

The NICE-2 study is designed as a three-arm, multicenter, prospective, randomized, phase II clinical trial, comparing camrelizumab plus chemotherapy (IO-CT) and camrelizumab plus CRT (IO-CRT) versus CRT as preoperative treatment for locally advanced ESCC. A total of 204 patients will be recruited from 8 Chinese institutions within 1.5 years. The primary endpoint is pathological complete response (pCR) rate and secondary endpoints include event-free survival (EFS), R0 resection rate, and adverse events.

**Discussion:**

This is the first prospective randomized controlled trial to explore commonly used neoadjuvant treatments in clinical practice, which will provide high-level evidence of neoadjuvant treatment for patients with locally advanced ESCC. The purpose of this study is to establish the optimal modality of IO-CT, IO-CRT and CRT as preoperative treatment for locally advanced ESCC. The Institution Review Committee approved this study protocol in August 2021 and patient enrollment was started in September 2021.

**Trial registration:**

ClinicalTrial.gov: NCT05043688 (August 29, 2021). The trial was prospectively registered.

## Background

Esophageal cancer remains the sixth most common cause of cancer-related death worldwide, and more than half of esophageal cancers were diagnosed as locally advanced disease [1]. A series of clinical trials have been conducted by scholars from all over the world to establish standard treatments for esophageal cancer in the past decades [2]. In the CROSS trial, prolonged overall survival had promoted the preoperative chemoradiotherapy (CRT) as recommended treatment for locally advanced esophageal cancer [3]. Despite the survival benefits, half of patients still relapsed after preoperative CRT and surgery, of whom distant metastasis was the most commonly observed [4]. This indicated that more effective systemic treatment is required to against potential micrometastases.

The role of immunotherapy, represented by PD-1 inhibitors, has been confirmed as the first-line treatment for advanced esophageal cancer by several landmark phase III trials including KEYNOTE-590 [5], CheckMate-648 [6] and ESCORT-1st [7]. Shortly afterwards, the participation of immunotherapy as neoadjuvant treatment has been widely explored in locally advanced esophageal cancer [8, 9]. Immunotherapy combined with CRT had been investigated in previous studies, such as the PALACE-1 study [10], in which 55.6% of patients achieved pathological complete response (pCR). However, 1 (5%) patient of the enrolled 20 patients died prior to surgery due to severe hemorrhage, as well as 12.9% mortality was observed in Korea’s study [8], which prompted us to concern for the treatment safety profile. Nevertheless, the combination of preoperative immunotherapy and CRT brings satisfactory short-term results. To date, immunotherapy combined with chemotherapy remains the preferred modality. A phase II study (NICE) [11], conducted by our group, achieved a 39.2% pCR rate and acceptable toxicity in locally advanced ESCC after preoperative camrelizumab plus weekly chemotherapy. The advantages including treatment convenience (short induction interval), well tolerability (high degree of treatment completion), and potential persisting systemic effect had been accepted. Therefore, it should be applied as a promising neoadjuvant treatment for esophageal cancer in the future.

Hence, we launched a three-arm randomized controlled trial to assess the superiority of camrelizumab plus chemotherapy (IO-CT) and the superiority of camrelizumab plus CRT (IO-CRT) in tumor response and long-term survival over CRT as preoperative treatment for locally advanced ESCC. It is the first prospective randomized controlled trial to compare the three common combinations of neoadjuvant treatments in patients with locally advanced ESCC. This study protocol has been approved by the Institution Review Committee in August 2021 (No. LS2160) and we started to recruit participants from September 2021. In each institution, approval by the institutional review board is obtained before starting patient accrual. This trial was registered at ClinicalTrials.gov (NCT05043688).

### Objective

The purpose is to assess the superiority of camrelizumab plus chemotherapy and the superiority of camrelizumab plus CRT in tumor response and long-term survival over CRT as preoperative treatment for locally advanced ESCC.

## Methods/design

### Study setting

**Fig. 1:**
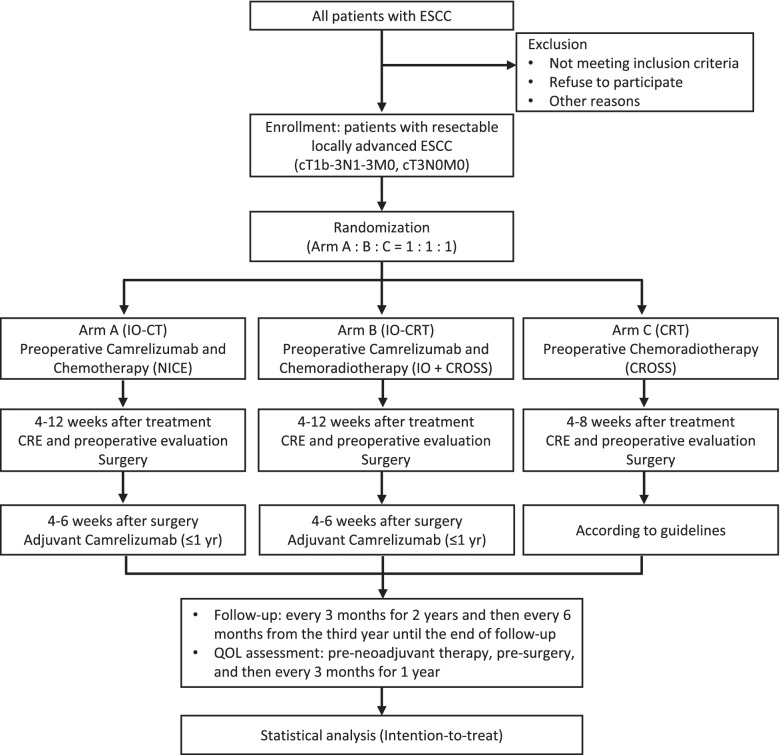
Flow chart of the NICE-2 study. ESCC: esophageal squamous cell carcinoma; IO: immunotherapy; CT: chemotherapy; CRT: chemoradiotherapy; CRE: clinical response evaluation; QOL: quality of life

This is a prospectively multicenter three-arm open label randomized Phase II study. The trial flow chart is presented in Fig. 1.

### Endpoints

The primary endpoint is pCR rate in all per-protocol patients. pCR is defined as the proportion of patients who had complete response of both primary tumor and metastatic lymph nodes in the resected specimen.

The secondary endpoints are event-free survival (EFS), R0 resection rate, and adverse events. EFS is defined as the duration from randomization to progression or death from any cause, and it is censored at the latest day the patient is alive without progression. Disease progression except for distant metastasis during preoperative therapy is not regarded as an event if R0 resection is conducted [12]. Adverse events are recorded including any adverse events during neoadjuvant treatment, surgical morbidity and mortality.

### Study population

Patients with locally advanced ESCC are eligible for enrollment in this trial. The primary investigator will take charge of the enrollment according to the inclusion/exclusion criteria.

#### Inclusion criteria

1. Histologically-confirmed squamous cell carcinoma.
2. Primary lesions are located in the thoracic esophagus.
3. Clinical stages as T1b-3N1-3M0 or T3N0M0 based on the 8th UICC-TNM classification.
4. 18–75 years of age.
5. ECOG performance status of 0 or 1.
6. Treatment-naïve for esophageal cancer.
7. No prior chemotherapy, radiotherapy or immunotherapy against any cancers.
8. With adequate organ function.
9. R0 resection is expected.
10. Written informed consent.

#### Exclusion criteria

1. Patients with second primary malignant disease.
2. Pregnant women or women preparing for pregnancy.
3. Patients with concurrent autoimmune disease or history of chronic autoimmune disease.
4. Patients who received corticosteroid (equivalent to prednisone of > 10 mg/day) within 14 days prior to the first day of drug administration.
5. Patients infected with HIV, or with active hepatitis B or C (HBV DNA ≥ 10^4^ copies/ml; HCV RNA ≥ 10^3^ copies/ml).
6. Patients with a history of pneumonitis or interstitial lung disease with clinical evidence, such as interstitial pneumonia and pulmonary fibrosis found by baseline CT scan;
7. Patients with known or concurrent bleeding disorders or other uncontrolled diseases who cannot receive surgical treatment.
8. Physical examination or clinical trial findings that could interfere with the results or put the patient at increased risk for treatment complications.
9. Patients with comorbidities (chronic pulmonary disease, poorly controlled hypertension, unstable angina, myocardial infarction within 6 months, unstable mental disorders requiring therapy).
10. Patients who are allergic to study drugs.
11. Patients who participated in other clinical trials within 30 days before enrollment.
12. Patients who are considered as not suitable for participation by the investigators.

### Randomization

Patients with locally advanced, histologically-confirmed ESCC are recruited. All patients are mandatory to receive endoscopy with biopsy, neck/chest/abdomen enhanced CT scan, upper gastrointestinal radiography and PET-CT. After confirmation of the eligibility criteria, patients are randomized with 1:1:1 allocation ratio to any of the three arms by minimization method balancing the arms with institution and clinical stage (II vs. III vs. IVa) by the central clinical research coordinator. The three arms consist of arm A (IO-CT, camrelizumab plus chemotherapy, NICE), arm B (IO-CRT, camrelizumab plus CRT), and arm C (CRT, CROSS). The intervention in this study is not blinded.

### Preoperative treatment

**Fig. 2:**
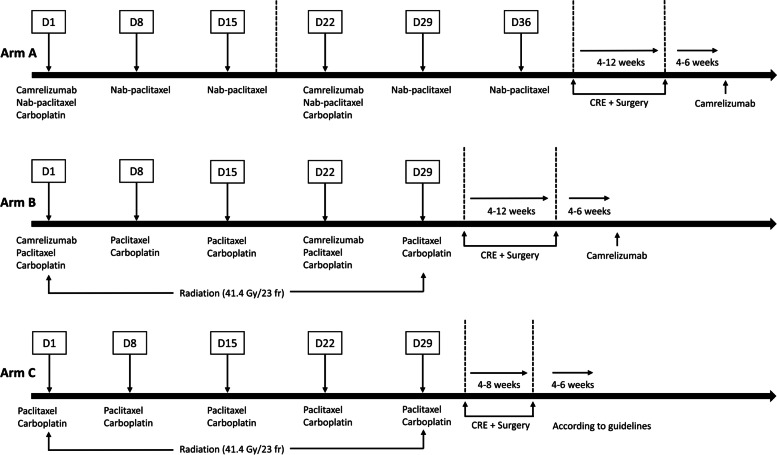
Neoadjuvant treatment plans for the three arms. CRE: clinical response evaluation

The detailed treatment regimens of three arms are presented in Fig. 2.

Patients in arm A receive two courses of preoperative camrelizumab and chemotherapy (camrelizumab, 200 mg/day, day 1; nab-paclitaxel, 100 mg/m^2^/day, day 1/8/15; carboplatin, area under the curve (AUC) of 5 mg/mL/min, day 1) repeated every 3 weeks [11].

Patients in arm B receive two courses of preoperative camrelizumab (200 mg/day, day 1) repeated every 3 weeks and CRT with CROSS regimen ie. five weekly cycles of carboplatin (AUC of 2 mg/mL/min) and paclitaxel (50 mg/m^2^/day, day 1/8/15/22/29) with concurrent radiotherapy (41.4 Gy in 23 fractions, 5 days per week) [3].

Patients in arm C receive preoperative CRT with CROSS regimen ie. five weekly cycles of carboplatin (AUC of 2 mg/mL/min) and paclitaxel (50 mg/m^2^/day, day 1/8/15/22/29) with concurrent radiotherapy (41.4 Gy in 23 fractions, 5 days per week) [3].

Reductions are not permitted for camrelizumab, but are permitted for chemotherapy in accordance with two levels of dosage in case of severe febrile neutropenia or neutropenia, thrombocytopenia and anemia. Treatment should be interrupted or delayed if a severe adverse event occurs, and could be resumed until the protocol-defined criteria for treatment resumption is met. Adverse events are recorded and graded according to Common Terminology Criteria for Adverse Event (CTCAE) v5.0 [13].

### Surgery

Approximately 4–6 weeks after neoadjuvant treatment, patients will be re-evaluated by the investigators according to the Response Evaluation Criteria for Solid Tumors (RECIST) 1.1. If there was no evidence of metastatic disease, esophagectomy will be performed. Minimally invasive esophagectomy (MIE), open right thoracotomy esophagectomy or hybrid surgery with at least 2-field lymphadenectomy is allowed. Either transhiatal or left thoracotomy esophagectomy is not acceptable due to the limited capacity for upper mediastinum lymph nodes dissection. Surgery-related complications will be recorded on the case report form for up to 90 days after surgery.

### Follow up

All randomized patients are followed up for at least 5 years while the analysis of primary endpoint is performed after surgery. Follow-up will be conducted every 3 months for the first 2 years, every 6 months for years 3–5, and then every year for life. Usually, the CT scan is performed every 3 months in the first year and every 6 months from the second year. Endoscopy is performed every 6 months in the first 2 years and every 12 months from the third year. Emission computed tomography (ECT) and brain magnetic resonance imaging (MRI) are performed every year.

### Translational research

This study will prospectively collect tissue and blood samples for translational research after the acquisition of additional written consent from participants. The expected tissue sample consists of a pre-treatment biopsy tumor tissue and normal mucosa, as well as tumor tissue and normal mucosa in surgical specimens. Blood samples should be obtained at the time of pre-treatment evaluations, during neoadjuvant therapy, prior to surgery, 3 weeks and 3 months after surgery.

### Statistical analysis

Considering the primary endpoint, we assumed pCR rate with preoperative CRT (arm C) in real-world Asia population to be 24.6% [14]. The pCR rate of preoperative camrelizumab and chemotherapy (arm A) was 39.2% in the phase II NICE study [11]. Based on the Schoenfeld and Richter’s method [15], the sample size was calculated as 61 patients per arm with a study-wise one-sided alpha level of 5%, a power of 80% for each pair-comparison, an expected accrual period of 1.5 years and a follow-up period of 3 years. Considering a dropout rate of 10%, each group needs to be enrolled in 68 subjects, and the total sample size was set at 204 patients. Only when the superiority of both arm A and arm B over arm C is confirmed, the direct comparison between arm A and arm B will be conducted with a one-sided alpha of 5% in a closed testing procedure.

### Interim analysis and monitoring

Interim analysis is scheduled after half of the planned number of patients has been enrolled. The Data and Safety Monitoring Committee (DSMC) will review the reports independently from the investigators and statistician. Data will be analyzed according to the intention-to-treat (ITT) principle with all randomized patients and per-protocol (PP) principle with all patients who sufficiently comply with the protocol. If the superiority of either one of the test arms is confirmed with an adjusted alpha level, the study will be continued with the other two arms. If the superiorities of both test arms are confirmed over the control arm, the study will be continued only with the two test arms. Active monitoring will be performed every 3 months by DSMC to improve study progress, ensure data integrity and patient safety.

### Current status

This study has been ethically approved by the ethics committees of Shanghai Chest Hospital in August 2021 (No. LS2160). Recruitment of patients was started in September, 2021. It is still at the stage of recruiting as 40 patients have been recruited until March 15, 2022.

## Discussion

Nowadays, standard neoadjuvant treatment for locally advanced esophageal cancer mainly includes chemoradiotherapy, chemotherapy, or emerging combination with immunotherapy. However, safety profiles and efficacy including survival benefits among diverse neoadjuvant treatments have not been well verified. Therefore, a head-to-head comparison among the above commonly used regimens is being pursued.

The ESCORT-1st study [7] was a phase III clinical trial conducted in Chinese population, which confirmed that camrelizumab plus chemotherapy could significantly prolong survival and it was well tolerated in the first-line treatment for advanced ESCC. Then, studies on camrelizumab plus chemotherapy as preoperative treatment for locally advanced ESCC have been investigated in several centers around China [16, 17]. Among them, the NICE study achieved the best pCR rate, which consists of two 3-week cycles of preoperative camrelizumab and chemotherapy with weekly nab-paclitaxel and high-dose of carboplatin (AUC = 5). So, preoperative camrelizumab plus chemotherapy in this phase II study demonstrated with robust and stable antitumor activity, as well as acceptable safety profile in locally advanced ESCC [11].

Moreover, immunotherapy plus CRT had also been assessed in several clinical trials [18, 19], including the PALACE-1 study [10] for squamous cell carcinoma and the PERFECT study [9] for adenocarcinoma. Dr. Kim et al. [8] firstly reported the combination of Pembrolizumab with CRT as preoperative treatment for locally advanced esophageal cancer. However, 12.9% of mortality has made the safety of this regimen as a concern, which was also disputed in the PALACE-1 study. According to a real-world multicenter retrospective study from China, immunotherapy plus chemotherapy remains the most commonly used combination modality, while immunotherapy plus CRT resulted in higher pCR rate as well as increased adverse events (data not published). Therefore, there has been no conclusive evidence supporting the comparison of different neoadjuvant treatments at present.

The present NICE-2 study compares preoperative immunotherapy plus chemotherapy and preoperative immunotherapy plus CRT with preoperative CRT followed by surgery, which was designed as control arm. Preoperative CRT followed by surgery is the most evidenced treatment modality for locally advanced ESCC in China. As preoperative CRT plus surgery has already been proven by two randomized controlled trials [3, 20] to be superior to surgery alone, it was designed as the control group. The preoperatively concurrent radiation with 41.4 Gy and carboplatin/paclitaxel (CROSS regimen) was chosen as the control intervention due to that it has been widely used not only in China but also internationally in Western Europe and North America.

## Conclusion

The NICE-2 study is a multicenter prospective randomized controlled trial, comparing preoperative camrelizumab plus chemotherapy (IO-CT) or camrelizumab plus CRT (IO-CRT) versus preoperative CRT with CROSS regimen (CRT) followed by surgery for the treatment of locally advanced ESCC. It is hypothesized that perioperative immunotherapy could result in prolonged survival based on comparable local tumor control and potential systemic effect for micrometastases.

## Data Availability

The datasets used during the NICE-2 study are available from the corresponding author on reasonable request.

## Abbreviations

CRT: Chemoradiotherapy
ESCC: Esophageal squamous cell carcinoma
PD-1: Programmed cell death protein 1
IO: Immunotherapy
pCR: Pathological complete response
EFS: Event-free survival
CRE: Clinical response evaluation
TNM: Tumor-Node-Metastasis
UICC: International Union for Cancer Control
ECOG: European Clinical Oncology Group
CT: Computed tomography
PET-CT: Positron emission tomography-computed tomography
ECT: Emission computed tomography
MRI: Magnetic resonance imaging
AUC: Area under the curve
CTCAE: Common Terminology Criteria for Adverse Event
RECIST: Response Evaluation Criteria for Solid Tumors
MIE: Minimally invasive esophagectomy
ITT: Intention-to-treat
PP: Per-protocol
DSMC: Data and Safety Monitoring Committee
